# Experimental Vitamin D Deficiency in Rats: Clinical Chemistry, Histopathological, and Immunological Evaluation

**DOI:** 10.7759/cureus.67490

**Published:** 2024-08-22

**Authors:** Mustafa Erinc Sitar, Yaprak Donmez Cakil, Belkiz Ongen Ipek, Necdet Altıner, Mehmet Serif Aydin, Hakan Gunal, Ali Diyar Atamis, Aslı Karadeniz

**Affiliations:** 1 Department of Medical Biochemistry, Maltepe University Faculty of Medicine, Istanbul, TUR; 2 Department of Histology and Embryology, Maltepe University Faculty of Medicine, Istanbul, TUR; 3 Experimental Animals Research and Application Center, Maltepe University, Istanbul, TUR; 4 Regenerative and Restorative Medicine Research Center, Istanbul Medipol University, Istanbul, TUR; 5 Department of Infectious Diseases, Maltepe University Faculty of Medicine, Istanbul, TUR

**Keywords:** vitamin d deficiency, immunophenotyping, histopathology, cytokines, clinical chemistry

## Abstract

Background: Vitamin D deficiency is a significant global health concern. Experimental models are essential to elucidate the biochemical, histopathological, and immunological consequences of this deficiency. This study established a vitamin D deficiency rat model to mimic insufficient vitamin D intake and examine the resulting health impacts, particularly on liver, kidney, and immune functions.

Materials and methods: Sprague-Dawley male rats were randomly assigned to two groups. The control group received a standard rodent diet, while the experimental group was fed a modified diet with reduced vitamin D for three months. Analyses included serum vitamin D levels, clinical chemistry, renal and liver histopathology, and blood immunophenotyping and cytokine analysis for both the control (n=7) and experimental (n=7) groups.

Results: Serum vitamin D 25-OH levels were threefold lower in the experimental group (p < 0.001), indicating the induction of vitamin D deficiency. No significant differences in weight gain were observed between the groups. All clinical chemistry parameters remained within reference ranges. However, the experimental group showed significant declines in triglycerides (TG, p=0.0441), alkaline phosphatase (ALP, p=0.0021), and alanine aminotransferase (ALT, p=0.0002). Histopathology revealed normal liver and kidney architecture in the control group, while the experimental group exhibited hepatic cord deterioration, severe vacuolization in the liver, and edema and dilatation in the renal cortex tubular epithelium. Immunophenotyping analysis of lymphocyte subsets and assessment of serum cytokines did not reveal any differences between the two groups.

Conclusion: A vitamin D deficiency model without complications such as obesity, parathyroid issues, or mortality was established in rats. This method could be applied in specific disease experimental models.

## Introduction

Vitamin D is a fat-soluble vitamin and a hormone precursor sterol that can be obtained from food sources of animal origin or, as a primary source, produced endogenously through exposure to ultraviolet-B (UV-B) sunlight on the skin. Following the initial UV-B-mediated reaction at the skin tissue level, the subsequent hepatic 25-hydroxylation and renal 1-alpha-hydroxylation reactions are essential for the full activation of this crucial vitamin. It is notable that subclinical vitamin D deficiency is prevalent in both developing and developed countries, with a worldwide prevalence of up to 1 billion people [1]. Deficiency of vitamin D results in the development of osteomalacia in adults and rickets in children. Although vitamin D primarily affects musculoskeletal tissues by modulating calcium and phosphorus metabolism, its receptors are distributed throughout the body. Consequently, subclinical deficiency of vitamin D has been linked to a wide range of systemic diseases, including chronic liver and renal diseases, malignancies, cardiovascular disorders, infections, and autoimmune conditions, in addition to directly related musculoskeletal complications such as osteoporosis, falls, and fractures [2,3]. Moreover, there is evidence that vitamin D levels may be a contributing factor in determining cause-specific or overall mortality [4,5].

Vitamin D is a pivotal mediator of the immune system, primarily through its anti-inflammatory and antimicrobial effects. It plays a multifaceted role in the regulation of both innate and adaptive immune responses [6]. A recent study examining inflammation in elderly patients with lower levels of vitamin D revealed an association between vitamin D deficiency and the incidence of inflammatory events [7]. A further study demonstrated that the intestinal barrier function is compromised in the offspring of mothers with vitamin D deficiency, which in turn leads to an inflammatory response and further disruption of the intestinal microbiota in mice [8].

The high prevalence of vitamin D deficiency, the possible overlap with liver and renal pathologies, and its other diverse clinical implications prompted us to develop a vitamin D deficiency rat model and investigate the biochemical, histopathological, and immunological aspects. The diet was formulated to maintain calcium and phosphorus homeostasis, lipid metabolism, and fat storage while restricting weight gain. A three-month diet period was performed to evaluate the effects of vitamin D deficiency in this rat model. This study aims to provide a model without complications such as obesity, parathyroid problems, and mortality.

## Materials and methods

Animals, feeding model, and ethics approval

Sprague Dawley male rats (6 months old, n=16) were obtained from Maltepe University Experimental Animals Application and Research Center (MUDEHAM), Istanbul, Turkey. The experimental protocol was approved by the Maltepe University Local Ethics Committee on Animal Experiments (MUHADYEK-2019.09.02). The rats were divided into two groups (n=8 for each group) and maintained in separate rooms for three months. While the control group consumed routine rodent food (ARDEN Araştırma & Deney, Ankara, Turkey), the experimental group consumed a vitamin D-reduced diet with 10 kcal% fat and carbohydrate, mainly as corn starch, to induce hypovitaminosis D (based on Product #D12450K, Research Diets, New Brunswick, New Jersey; obtained from ARDEN Araştırma & Deney, Turkey). The experimental group animals were kept in partial darkness for four to six hours per day. Otherwise, all animals were housed in temperature-controlled rooms (25 ± 3 °C) with automatic 12-hour light/dark cycle periods. The weights of the animals were recorded routinely every week. The food and water for both the control and experimental groups were presented "ad libitum" and were checked regularly every day. Two rats in total (one from each group) were excluded from the study upon the decision of the veterinarian and animal welfare unit.

Vitamin D status

Intracardiac blood withdrawal was performed on anesthetized animals. Blood specimens were collected into appropriate serum test tubes and incubated for about 20 minutes to allow clot formation. Subsequently, the blood test tubes were centrifuged at 1500 g for 15 minutes, resulting in the separation of the supernatant fraction, or serum. Serum vitamin D levels were measured using the immunoassay method on the Abbott Architect (Abbott Park, Illinois) device. Following the completion of the routine daily internal quality control procedure, the measurements were taken in accordance with the manufacturer's instructions. The calibration status of the test was appropriate, and external quality controls were also monitored monthly.

Clinical chemistry analysis

The serum samples were divided into equal aliquots and stored at -80°C until further analysis. Prior to the measurements, two levels of internal quality control were performed. Routine clinical biochemistry assays were conducted on a Siemens Dimension Rxl (Munich, Germany) clinical chemistry device, following the manufacturer's instructions.

Histopathological evaluation of liver and kidney

The animals were euthanized due to hypovolemia following the acquisition of blood samples under deep anesthesia. The liver and kidney tissues were washed with ice-cold phosphate buffer solution (PBS), weighed, and preserved in 10% neutral buffered formalin. Tissue samples were fixed in 10% neutral buffered formalin, passed through a series of increasing alcohol concentrations (70%, 90%, 96%, and 100%) and xylene, and embedded in paraffin. Sections of 5-µm thickness were taken with a microtome, stained with hematoxylin and eosin (H&E) for histopathological evaluation, and examined under a light microscope (AxioZoom, Carl Zeiss, Germany).

Liver tissue was evaluated microscopically for interstitial edema, sinusoid congestion, hepatocellular necrosis, and hepatocyte vacuolization. Additionally, kidney tissue was evaluated microscopically for interstitial edema, epithelial changes, tubular degeneration, and capillary congestion. Each evaluation parameter was scored from 0 to 4 based on the microscopic coverage of the injury, and the scores were combined for each organ to obtain the histopathological score for each animal, according to Kubiak et al. [9].

Evaluation of lymphocyte subpopulations

Two milliliters of rat peripheral blood plus ethylenediaminetetraacetic acid (EDTA) was diluted in PBS in a 1:1 ratio and gently layered on 2 mL of Histopaque (1.077 g/mL, Sigma-Aldrich, St. Louis, Missouri). Peripheral blood mononuclear cells (PBMCs) were isolated by density centrifugation at 400 g for 40 minutes at room temperature. The buffy coat was then carefully collected and washed twice with PBS containing 1% fetal bovine serum (FBS). After centrifugation for five minutes at 400 g, the pellet was obtained and resuspended in PBS containing 1% FBS for labeling with antibodies. Anti-rat CD-3 APC, anti-rat CD45RA FITC, anti-rat CD161a PE, and rat T lymphocyte cocktail (BD Biosciences, San Jose, California) antibodies were used for the identification of T, B, natural killer (NK), and T cell subsets, respectively. Appropriate isotype controls were purchased and used. Following the antibody incubations for 30 minutes at room temperature, cells were washed twice and resuspended for analysis in a BD Accuri C6+® (BD Biosciences) flow cytometer.

Assessment of serum cytokines

Cytokine assessments were conducted using serum samples. The concentrations of interleukin-2 (IL-2), IL-4, IL-1α, IL-10, interferon-gamma (IFN-γ), and tumor necrosis factor (TNF) were measured using the Cytometric Bead Array (CBA) Mouse/Rat Soluble Protein Master Buffer Kit (BD Biosciences) and the respective Rat Flex Sets (BD Biosciences). The procedure is based on the use of six bead populations coated with specific capturing antibodies, which exhibit distinct fluorescence intensities for the detection of each cytokine. The theoretical limit of detection was provided as follows: 0.46 pg/mL for IL-2, 3.4 pg/mL for IL-4, 4.0 pg/mL for IL-1α, 19.4 pg/mL for IL-10, 27.7 pg/mL for TNF, and 6.8 pg/mL for IFN-γ. We followed the instructions provided by the manufacturers. The assay procedure involved preparing serially diluted standards, incubation with capture beads, and subsequent incubation with PE detection reagent. Fifty microliters of each unknown sample was used for the assay. Detection was performed using a BD Accuri C6+® flow cytometer. The reporter channel was FL-2, and the bead channels were FL-3 and FL-4.

Statistical analysis

All data are expressed as mean ± SD. The Shapiro-Wilk test was performed to assess the normality of the distribution, and the difference between the control and experimental groups was evaluated using either the Student’s t-test or the Mann-Whitney U test with GraphPad Prism V.8.01 (GraphPad Software, San Diego, California). A p-value of <0.05 was considered statistically significant.

## Results

Body weights

**Figure 1:**
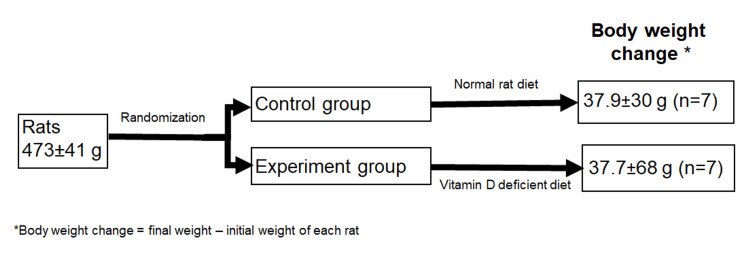
Body weights (g) and body weight changes of control and vitamin D-deficient (experiment) Sprague Dawley male rats over the experimental period. Values are expressed as mean ± standard deviation (SD). Mann-Whitney U test was performed to evaluate the statistical difference.

Sprague Dawley male rats were randomly divided into two groups: control and experimental. Initial and final body weights, as well as body weight changes of male rats in both control and experimental groups, are depicted in Figure 1. All animals gained weight over the experimental period. Body weight changes between the two groups were found to be similar (37.9±30.8 vs. 37.7±68.4, p>0.9999).

Induction of vitamin D deficiency

**Figure 2:**
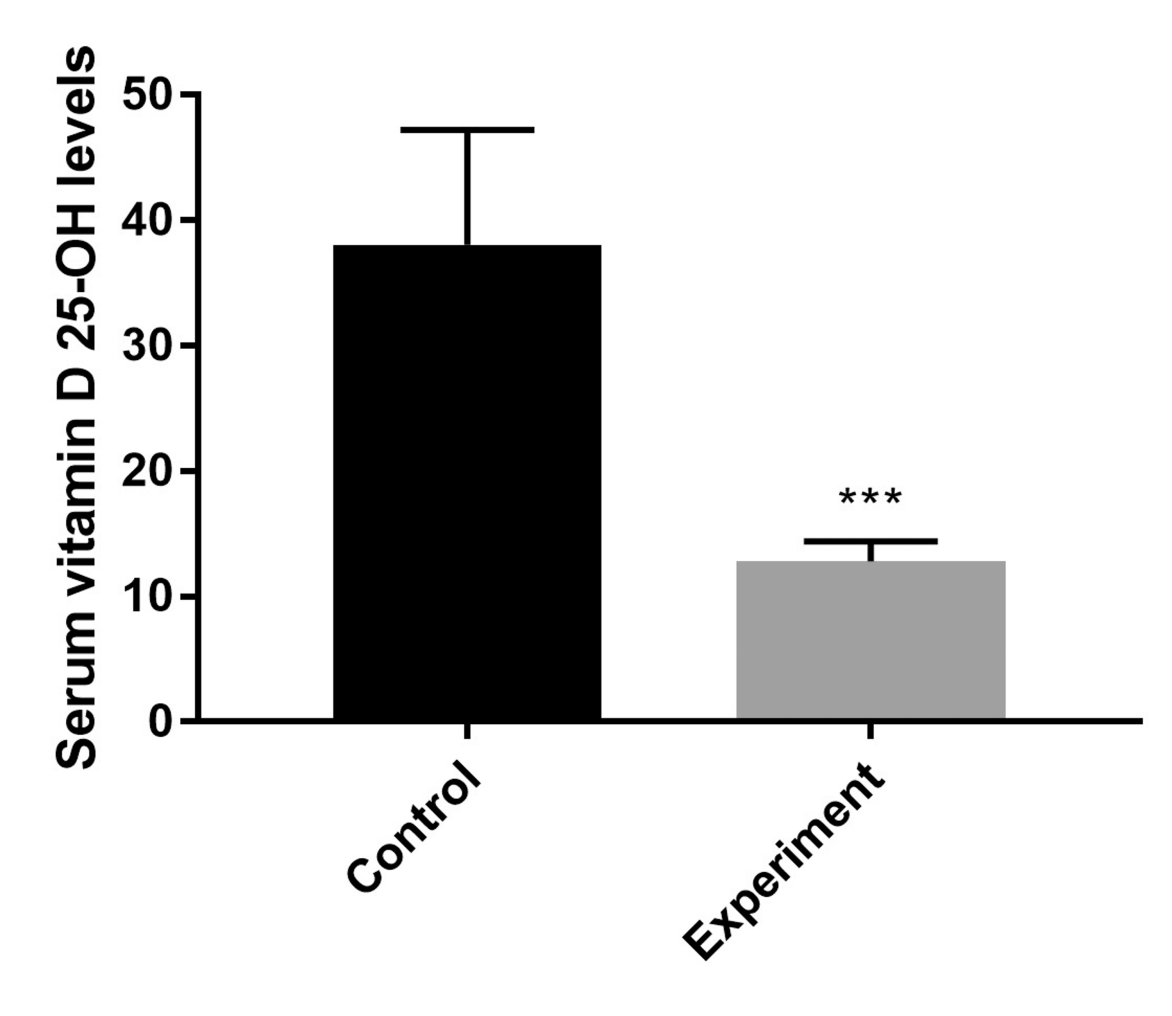
Final serum vitamin D 25-OH levels of control and vitamin D-deficient (experiment) Sprague Dawley male rats (ng/mL). Values are expressed as mean ± SD. Student’s t-test was performed to evaluate the statistical difference. The mean vitamin D 25-OH level of the experimental group was significantly different from that of the control group (***p<0.001).

As shown in Figure 2, final serum vitamin D 25-OH levels were measured at 38.1 ± 9.1 ng/mL and 12.8 ± 1.6 ng/mL in the control and experimental groups, respectively (p<0.001), indicating the development of vitamin D deficiency in the experimental group.

Histopathological observations

**Figure 3:**
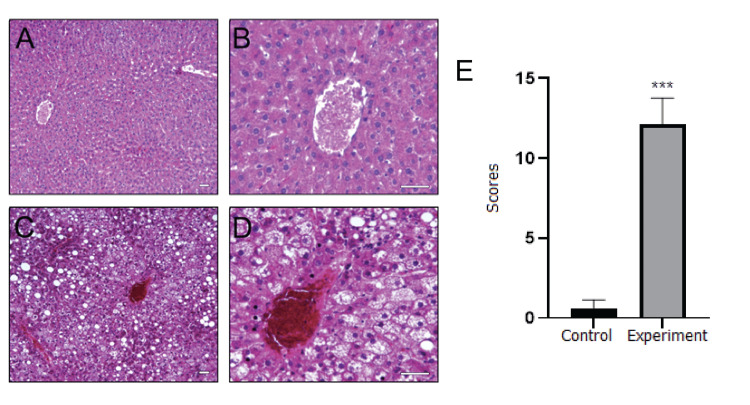
Microscopic images of the liver for control (A-B) and experimental (C-D) groups. Histopathological evaluation scores of control and vitamin D-deficient (experiment) Sprague Dawley male rats (E). The central vein located in the center of the classical liver lobule is seen in both groups. While regular hepatic cords around the central vein were observed in the control group, hepatic cord deterioration, severe vacuolization, congestion, and necrosis in hepatocytes were observed in the experimental group (hematoxylin and eosin staining, bar: 50 µm, A and C x125, B and D x400). Values are expressed as mean ± SD. ***Significantly different from control at P<0.001. The Mann-Whitney U test was performed to evaluate the statistical difference.

**Figure 4:**
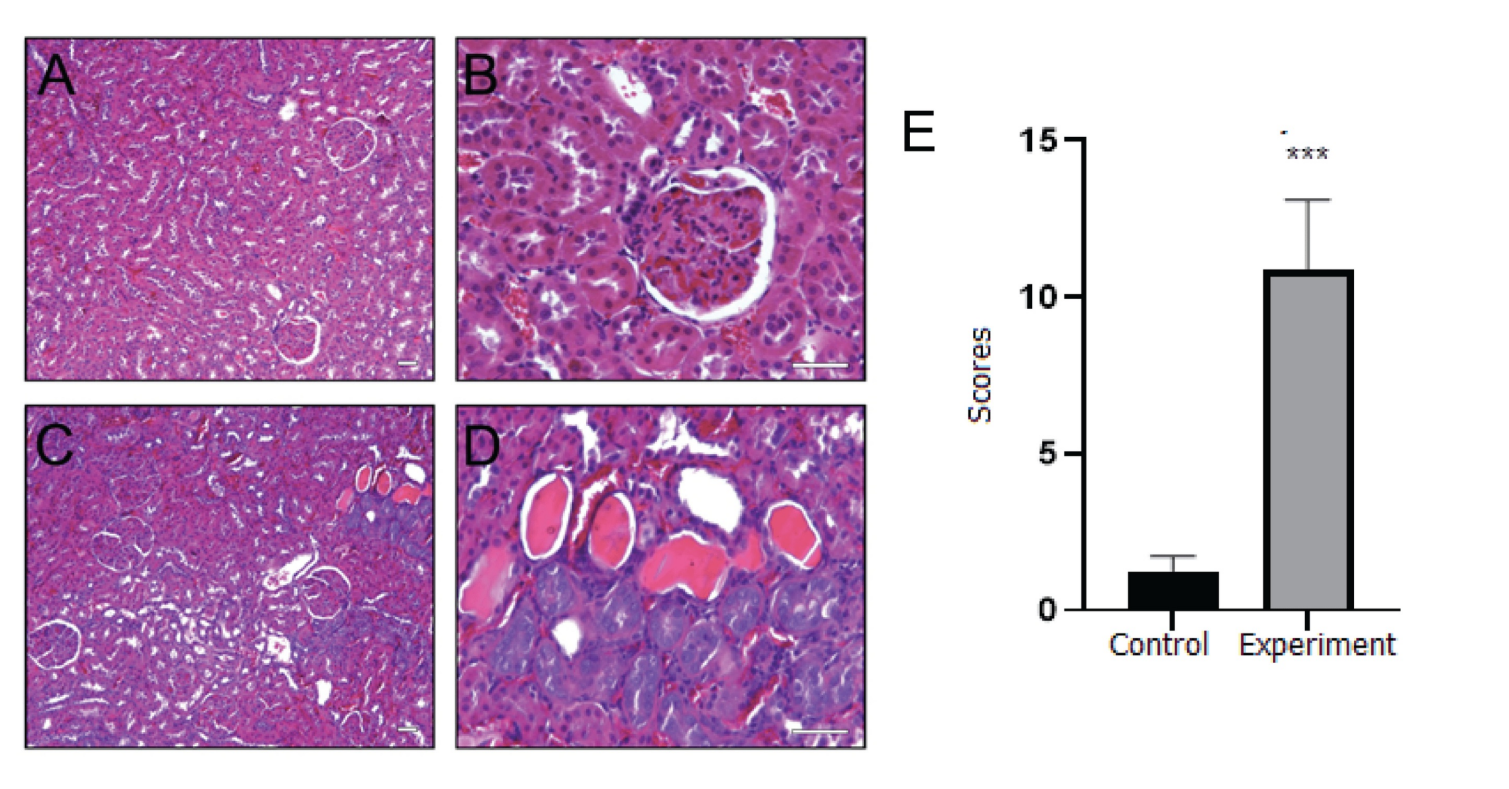
Microscopic images of the kidney for control (A-B) and experimental (C-D) groups. Histopathological evaluation scores of control and vitamin D-deficient (experiment) Sprague Dawley male rats (E). In both groups, glomeruli, proximal, and distal tubules are visible in the renal cortex region. While the glomeruli and tubules exhibited normal morphology in the control group, dilatation, edema, shedding, and congestion were observed in the tubular epithelium in the experimental group (hematoxylin & eosin staining, bar: 50 µm, A and C x125, B and D x400). Values are expressed as mean ± SD. ***Significantly different from control at P<0.001. The Mann-Whitney U test was performed to evaluate the statistical difference.

Figures 3, 4 demonstrate the histopathological alterations and evaluation scores of the liver and kidney, respectively, in the control (A and B) and vitamin D-deficient (experimental group, C and D) rats. The livers of the control group displayed a normal microscopic architecture with the central vein located in the center of the classical liver lobule (Figures 3A and B). In contrast, vitamin D deficiency resulted in hepatic cord deterioration, severe vacuolization, congestion, and necrosis in hepatocytes, as evident in Figures 3C and D. Histopathological evaluation scores of hepatic tissue were obtained by summing the score of each parameter (Figure 3E) and were found to be significantly higher in the experimental group compared to the control group (p = 0.0002).

Figure 4 demonstrates the histological alterations in the renal cortex of vitamin D-deficient rats. While normal histology of the rat renal cortex, with glomeruli and tubules, was observed in the control group (Figures 4A and B), dilatation, edema, shedding, and congestion were found in the tubular epithelium of the experimental group (Figures 4C and D). The mean histopathological evaluation score of renal tissue was significantly higher in the experimental group compared to the control group (p = 0.0002, Figure 4E).

Serum biochemical parameters

Comparison of clinical biochemistry parameters from serum in the control and experimental groups revealed significant differences in the levels of triglycerides, alkaline phosphatase (ALP), and alanine aminotransferase (ALT), as depicted in Table 1. These parameters were significantly lower in vitamin D-deficient rats compared to control rats (p=0.0441 for triglycerides, p=0.0021 for ALP, and p=0.0002 for ALT). The measured levels of the other examined parameters, namely total cholesterol, low-density lipoprotein (LDL), high-density lipoprotein (HDL), gamma-glutamyl transferase (GGT), total protein, creatinine, aspartate aminotransferase (AST), phosphorus, and calcium, were similar between the two groups (p>0.05).

**Table 1:** Serum biochemistry parameters in control and vitamin D-deficient (experiment) Sprague Dawley male rats. Triglyceride (p=0.0441), alkaline phosphatase (ALP, p=0.0021), and alanine aminotransferase (ALT, p=0.0002) values were significantly higher in the control group compared to the experimental group. Values are expressed as mean ± SD, and the p-value is given for each parameter. *Significantly different from control at p<0.05, **significantly different from control at p<0.01, ***significantly different from control at p<0.001, ****significantly different from control at p<0.0001. Student’s t-test was performed to evaluate the statistical difference.

	Control	Experiment	p-value
Triglyceride (mg/dL)	304 ± 115.5	196.7 ± 50.9	0.0441*
Total cholesterol (mg/dL)	125.1 ± 28.7	135.7 ± 30.1	0.5143
Low-density lipoprotein (LDL, mg/dL)	19.1 ± 5.2	19.4 ± 3.9	0.9092
High-density lipoprotein (HDL, mg/dL)	84.1 ± 18.2	92.6 ± 19.6	0.4206
Gamma-glutamyl transferase (GGT, U/L)	5 ± 0.8	5 ± 0.6	0.999
Alkaline phosphatase (ALP, U/L)	155.1 ± 24.7	107 ± 21.4	0.0021**
Total protein (g/dL)	6.5 ± 0.4	7 ± 1	0.375
Creatinine (mg/dL)	0.44 ± 0.2	0.5 ± 0.1	0.7184
Aspartate aminotransferase (AST, U/L)	157.7 ± 25	161.1 ± 67.2	0.9014
Alanine aminotransferase (ALT, U/L)	75.5 ± 17.4	38.4 ± 5.9	0.0002***
Phosphorus (mg/dL)	5 ± 1.9	7.6 ± 2.4	0.7751
Calcium (mg/dL)	10.5 ± 4	11.4 ± 1.2	0.6562

Immunophenotyping analysis of peripheral blood

Lymphocyte subpopulations were analyzed to assess the immune status of vitamin D-deficient rats (Table 2). Antibodies targeting T cells, B cells, NK cells, and T cell subtypes were employed. No statistical difference was found between the control and experimental groups in the percentages of T cells (CD3+), B cells (CD45RA+), NK cells (CD161a+), T helper cells (CD3+CD4+), T cytotoxic cells (CD3+CD8+), or the T helper/T cytotoxic ratio.

**Table 2:** Immunophenotyping analysis of peripheral blood (expressed as %) of the control and vitamin D-deficient (experiment) Sprague Dawley male rats. Values are expressed as mean ± SD.  Student’s t-test was performed to evaluate the statistical difference.

	Control	Experiment	p-value
T cells	49.7 ± 7.7	49.1 ± 5.9	0.8262
T helper cells	75.9 ± 8.0	74.0 ± 4.8	0.5923
T cytotoxic cells	22.4 ± 8.0	24.2 ± 4.8	0.6045
T helper/T cytotoxic	4.0 ± 2.1	3.2 ± 0.9	0.3735
B cells	27.3 ± 7.2	23.3 ± 3.1	0.1993
Natural killer (NK) cells	4.5 ± 1.1	4.4 ± 1.9	0.9194

Serum cytokine profiles

Serum cytokine levels were determined to assess whether vitamin D status influences immune system function without any stimulation process (Table 3). The levels of IL-2, IL-4, IL-1α, IL-10, IFN-γ, and TNF were compared, and no significant changes were found between the control and experimental groups (p=0.4461, p=0.0979, p=0.7546, p=0.9452, p=0.5483, and p=0.6092, respectively).

**Table 3:** Serum cytokine concentrations (pg/mL) in the control and vitamin D-deficient (experiment) Sprague Dawley male rats. Values are expressed as mean ± SD. ^a^Student’s t-test. ^b^Mann-Whitney U test. IL: interleukin, IFN-γ: interferon-gamma, TNF: tumor necrosis factor.

	Control	Experiment	p-value
Interleukin-2^a^	6.9 ± 6.6	4.5 ± 4.5	0.4461
Interleukin-4^a^	13.7 ± 8.4	31.6 ± 23.21	0.0979
Interleukin-1α^b^	65.2 ± 44.5	52.4 ± 36.2	0.7546
Interleukin-10^b^	83.2 ±54.4	95.6 ± 65.6	0.9452
Interferon-gamma^a^	7.4 ±1.3	7.2 ± 0.5	0.5483
Tumor necrosis factor^a^	30.9 ±6.7	32.6 ± 5.5	0.6092

## Discussion

Insufficiency or deficiency of vitamin D is a global health problem associated with multiple comorbidities across nearly all age groups, primarily due to limited natural dietary sources of vitamin D and insufficient sun exposure. The current medical literature contains many controversial publications regarding vitamin D deficiency, its replacement, its relationship with diseases, and its impact on general health. To reduce the risk of various diseases associated with hypovitaminosis D, researchers are currently working on predictive models for vitamin D deficiency based on vitamin D status [10].

In the current study, the aim was to establish a vitamin D deficiency model in Sprague Dawley male rats to investigate the impact primarily on liver, kidney, and immune functions. Weight gains were found to be similar over the experimental period between the control and vitamin D-deficient rats. Various models of hypovitaminosis D have been published, with some reporting no change [11,12] and others a decrease [13-15] in weight gain in vitamin D-deficient rats or mice, depending on the protocol of induction. On the other hand, when the rats were fed a vitamin D-reduced diet that was high in dietary fat and contained high-fructose corn syrup, a diet-induced obesity combined with vitamin D deficiency was observed [16]. In our study, while the body weights of the two groups did not differ, triglyceride values were significantly lower in the vitamin D-deficient group. Since triglyceride values were within the reference ranges for the rats, this finding is thought to be related to the altered liver function test results, as explained below.

A threefold decrease in serum vitamin D 25-OH levels was demonstrated in the experimental group, corresponding to the induction of vitamin D deficiency. However, total protein values, which indicate hepatocyte synthesis capacity, were not statistically different between the groups and were within the reference ranges in both groups [17]. AST, ALT, and ALP activities are frequently used to assess liver functions and can identify potential damage to the liver as well as the biliary tract. ALP evaluation can also be useful in assessing bone diseases. Although ALP and ALT activities were within the reference ranges (21-367 IU/L and 6-114 IU/L, respectively) [17] in both groups, enzyme activities were lower in the experimental group. However, no change was detected in AST activity (reference range: 37-205 IU/L) [18]. Differences in ALP and ALT enzyme activities suggest changes in hepatocyte function in the liver due to (i) diet, (ii) diet-induced vitamin D insufficiency, or (iii) both, as discussed previously [15]. This finding is consistent with the histopathological data of the liver.

One of the most significant outcomes of the present study is the absence of hyperparathyroidism, which is commonly observed in vitamin D deficiency models. In this study, calcium and inorganic phosphorus values were within the reference ranges (9.5-11.3 mg/dL for calcium, 4.3-10.1 mg/dL for inorganic phosphorus) [17] in both groups, with no significant difference between them. This finding provides a positive indication of the animals’ bone health, in contrast to results obtained with other methods [11]. The level of creatinine, which is a product of muscle metabolism and indicates kidney function capacity, was also within the reference range, with no difference observed between the groups.

In the lipid panel, which included triglycerides, total cholesterol, LDL, and HDL, all measured values were within the reference ranges. Except for triglycerides, no significant differences were found between the groups. The difference in triglyceride levels may be attributed to variations in dietary intake, deterioration in liver function, and/or changes in very low-density lipoprotein (VLDL) metabolism. Again, it should be noted that triglyceride values were within the reference range.

Potential liver damage was indicated by histopathological observations, including deterioration of the hepatic cord, severe vacuolization, congestion, and necrosis in hepatocytes, as well as biochemical analysis in the experimental group. Similarly, Pahuja et al. [15] reported alterations in liver histology, indicating changes in liver function associated with vitamin D deficiency. While Bingül et al. [19] did not observe any changes in histopathological findings, they measured significant increases in hepatic triglyceride and tumor necrosis factor-alpha (TNF-α) levels and myeloperoxidase (MPO) activity, pointing to inflammation in the liver. Roth et al. [16] reported that vitamin D deficiency was not associated with further changes in liver histology when rats were fed a low-fat diet with reduced levels of vitamin D, compared to those fed only a low-fat diet. The researchers only observed increased hepatic steatosis and lobular inflammation when the rats were fed a high-fat diet with vitamin D depletion. Regarding the kidneys, dilatation, edema, leukocyte infiltration, shedding, and congestion were observed in the tubular epithelium, indicating a risk of kidney function impairment and inflammation. Rangan et al. [20] also suggested that vitamin D deficiency has adverse effects on the kidneys. They specifically focused on polycystic kidney disease in rats and demonstrated the differential and stage-specific effects of vitamin D on renal function.

Immunophenotyping analysis of peripheral blood allows for the examination of specific features of adaptive and innate immune function. Determining the immunophenotypic characteristics of T, B, and NK cells by evaluating the related cell-surface markers enables the characterization of immune responses in states of health and disease, including immunodeficiencies, autoimmunity, infections, and cancer [21]. Our study revealed no changes in the percentages of lymphocyte subpopulations, including T cells, B cells, NK cells, and T cell subsets-namely T helper and T cytotoxic cells-between the two groups, indicating no effect of long-term vitamin D deficiency on the proportions of lymphocyte subsets. The proportions of the lymphocyte subsets were similar to those reported by Ridge et al. [22], who employed Crl:WI(Han) and Crl:CD(SD) rats at 2, 4, and 7 months of age. Studies in humans have clearly demonstrated variation in the concentrations and proportions of different lymphocyte subpopulations with age and sex [23]. Serum cytokine levels also did not differ between the control and experimental groups, indicating similar spontaneous cytokine production in both groups. The relationship between vitamin D deficiency and levels of IL-1β, IL-4, and IL-6 was previously shown in obese rats fed a vitamin D-reduced high-fat diet [16]. Moreover, in humans, elevated vitamin D3 levels were shown to be associated with repressed lipopolysaccharide (LPS)-mediated production of IL-1β, IL-6, TNF-α, IFN-γ, and IL-10 during the summer [24]. Similar effects of vitamin D have also been reported in the context of bacterial infection [25]. To demonstrate spontaneous cytokine production, the current study did not employ any stimulatory reagents. Different responses might be observed if a stimulating agent were used.

The limitations of the study include the lack of musculoskeletal assessments, such as measurement of bone density and tendon strain, and the use of only male rats. While the liver enzyme activities observed in this study fall within the reference range, further investigation is required to elucidate the impact of dietary interventions on the molecular mechanisms governing lipid metabolism.

## Conclusions

The final immunoassay results of the study indicated a significant decrease in vitamin D levels, with a threefold difference compared to the control group. No obvious morphological or musculoskeletal anomalies were identified in the animals. Although biochemical and immunological data were within the reference range, histological changes were observed in rats with vitamin D deficiency. ALT, ALP enzyme activities, and triglyceride levels, which were within the reference range but showed statistical differences between the control and experimental groups, suggest that the condition would have become more detrimental if the dietary process had continued. Despite the aforementioned limitations, the absence of obesity and hyperparathyroidism in the animals represents a significant strength of this study. A vitamin D deficiency model was established in rats, which could be employed in specific disease experimental models. Additionally, the responses to different vitamin D replacement routes and doses in these diseases can be readily monitored.

## References

[1] Sizar O, Khare S, Goyal A, Givler A (2024). Vitamin D deficiency. StatPearls [Internet].

[2] Chang SW, Lee HC (2019). Vitamin D and health - the missing vitamin in humans. Pediatr Neonatol.

[3] Riccio P (2024). Vitamin D, the Sunshine molecule that makes us strong: what does its current global deficiency imply?. Nutrients.

[4] Dai L, Liu M, Chen L (2021). Association of serum 25-hydroxyvitamin D concentrations with all-cause and cause-specific mortality among adult patients with existing cardiovascular disease. Front Nutr.

[5] Völker D, Grünhage F, Wagenpfeil S, Lammert F, Stokes CS (2019). Serum 25-hydroxyvitamin D levels and mortality risk in patients with liver cirrhosis: a protocol for a systematic review and meta-analysis of observational studies. Syst Rev.

[6] Bui L, Zhu Z, Hawkins S, Cortez-Resendiz A, Bellon A (2021). Vitamin D regulation of the immune system and its implications for COVID-19: a mini review. SAGE Open Med.

[7] Alharbi SS, Albalawi AA Sr, Al Madshush AM (2024). Association between lower levels of vitamin D and inflammation in the geriatric population: a systematic review and meta-analysis. Cureus.

[8] Li P, Wang Y, Li P (2023). Maternal vitamin D deficiency aggravates the dysbiosis of gut microbiota by affecting intestinal barrier function and inflammation in obese male offspring mice. Nutrition.

[9] Kubiak BD, Albert SP, Gatto LA (2010). Peritoneal negative pressure therapy prevents multiple organ injury in a chronic porcine sepsis and ischemia/reperfusion model. Shock.

[10] Kuwabara A, Nakatani E, Tsugawa N (2022). Development of a predictive model for vitamin D deficiency based on the vitamin D status in young Japanese women: a study protocol. PLoS One.

[11] Stavenuiter AW, Arcidiacono MV, Ferrantelli E (2015). A novel rat model of vitamin D deficiency: safe and rapid induction of vitamin D and calcitriol deficiency without hyperparathyroidism. Biomed Res Int.

[12] Mallya SM, Corrado KR, Saria EA (2016). Modeling vitamin D insufficiency and moderate deficiency in adult mice via dietary cholecalciferol restriction. Endocr Res.

[13] Bingül İ, Aydın AF, Küçükgergin C, Doğan-Ekici I, Doğru-Abbasoğlu S, Uysal M (2021). The effect of 1,25-dihydroxyvitamin D3 on liver damage, oxidative stress, and advanced glycation end products in experimental nonalcoholic- and alcoholic- fatty liver disease. Turk J Med Sci.

[14] Lagishetty V, Misharin AV, Liu NQ (2010). Vitamin D deficiency in mice impairs colonic antibacterial activity and predisposes to colitis. Endocrinology.

[15] Pahuja DN, Deshpande UR, Soman CS, Nadkarni GD (1989). Altered hepatic function in vitamin D-deprived rats. J Hepatol.

[16] Roth CL, Elfers CT, Figlewicz DP (2012). Vitamin D deficiency in obese rats exacerbates nonalcoholic fatty liver disease and increases hepatic resistin and Toll-like receptor activation. Hepatology.

[17] Loeb WF, Quimby FW (1989). The Clinical Chemistry of Laboratory Animals. https://www.cabidigitallibrary.org/doi/full/10.5555/19892289118.

[18] Suckow MA, Weisbroth SH, Franklin CL (2005). The Laboratory Rat. https://experts.umn.edu/en/publications/the-laboratory-rat-2.

[19] Bingül İ, Aydın F, Kucukgergin C, Doğan Ekici AI, Dogru-Abbasoglu S, Uysal M (2021). The effect of vitamin D deficiency and 1,25(OH)2D3 treatment on oxidative stress and Nrf2-antioxidant signaling in ethanol-induced hepatotoxicity. Arch Clin Exp Med.

[20] Rangan GK, Schwensen KG, Foster SL, Korgaonkar MS, Peduto A, Harris DC (2013). Chronic effects of dietary vitamin D deficiency without increased calcium supplementation on the progression of experimental polycystic kidney disease. Am J Physiol Renal Physiol.

[21] Kokuina E, Breff-Fonseca MC, Villegas-Valverde CA, Mora-Díaz I (2019). Normal values of T, B and NK Lymphocyte subpopulations in peripheral blood of healthy Cuban adults. MEDICC Rev.

[22] Ridge K, Downes N, Finney B (2019). Effects of strain, sex and age on immunophenotyping parameters in the rat and mouse. Comp Clin Path.

[23] Melzer S, Zachariae S, Bocsi J, Engel C, Löffler M, Tárnok A (2015). Reference intervals for leukocyte subsets in adults: results from a population-based study using 10-color flow cytometry. Cytometry B Clin Cytom.

[24] Khoo AL, Chai LY, Koenen HJ, Sweep FC, Joosten I, Netea MG, van der Ven AJ (2011). Regulation of cytokine responses by seasonality of vitamin D status in healthy individuals. Clin Exp Immunol.

[25] Hoe E, Nathanielsz J, Toh ZQ (2016). Anti-inflammatory effects of vitamin D on human immune cells in the context of bacterial infection. Nutrients.

